# Microblog sentiment analysis using social and topic context

**DOI:** 10.1371/journal.pone.0191163

**Published:** 2018-02-02

**Authors:** Xiaomei Zou, Jing Yang, Jianpei Zhang

**Affiliations:** School of Computer Science and Technology, Harbin Engineering University, Harbin, Heilongjiang, China; Dalian University of Technology, CHINA

## Abstract

Analyzing massive user-generated microblogs is very crucial in many fields, attracting many researchers to study. However, it is very challenging to process such noisy and short microblogs. Most prior works only use texts to identify sentiment polarity and assume that microblogs are independent and identically distributed, which ignore microblogs are networked data. Therefore, their performance is not usually satisfactory. Inspired by two sociological theories (sentimental consistency and emotional contagion), in this paper, we propose a new method combining social context and topic context to analyze microblog sentiment. In particular, different from previous work using direct user relations, we introduce structure similarity context into social contexts and propose a method to measure structure similarity. In addition, we also introduce topic context to model the semantic relations between microblogs. Social context and topic context are combined by the Laplacian matrix of the graph built by these contexts and Laplacian regularization are added into the microblog sentiment analysis model. Experimental results on two real Twitter datasets demonstrate that our proposed model can outperform baseline methods consistently and significantly.

## Introduction

It is a very challenging task to get users’ real sentiment from large collections of short user-generated social media contents (e.g. microblogs). It is also of great value and has a wide range of application prospects to mining users’ sentiment, such as customer relationship management, recommendation systems, and business intelligence [1–3]. The automatic sentiment analysis task usually requires the machine to have a deep understanding of natural language [4], which has achieved some satisfactory performances in long formal text sentiment analysis [5–8]. However, its performance drops sharply when it is applied to microblog sentiment analysis as it assumes that texts are independent and identically distributed (i.i.d.). Compared with long formal texts, microblogs are much shorter and have various expression style, e.g., ‘lol’ and ‘It is so coooooooool!’, which exacerbates the problem of vocabulary sparsity. On the other hand, social media provides different types of metadata, such as user relations, which can be leveraged to improve the accuracy of microblog sentiment analysis.

**Fig 1:**
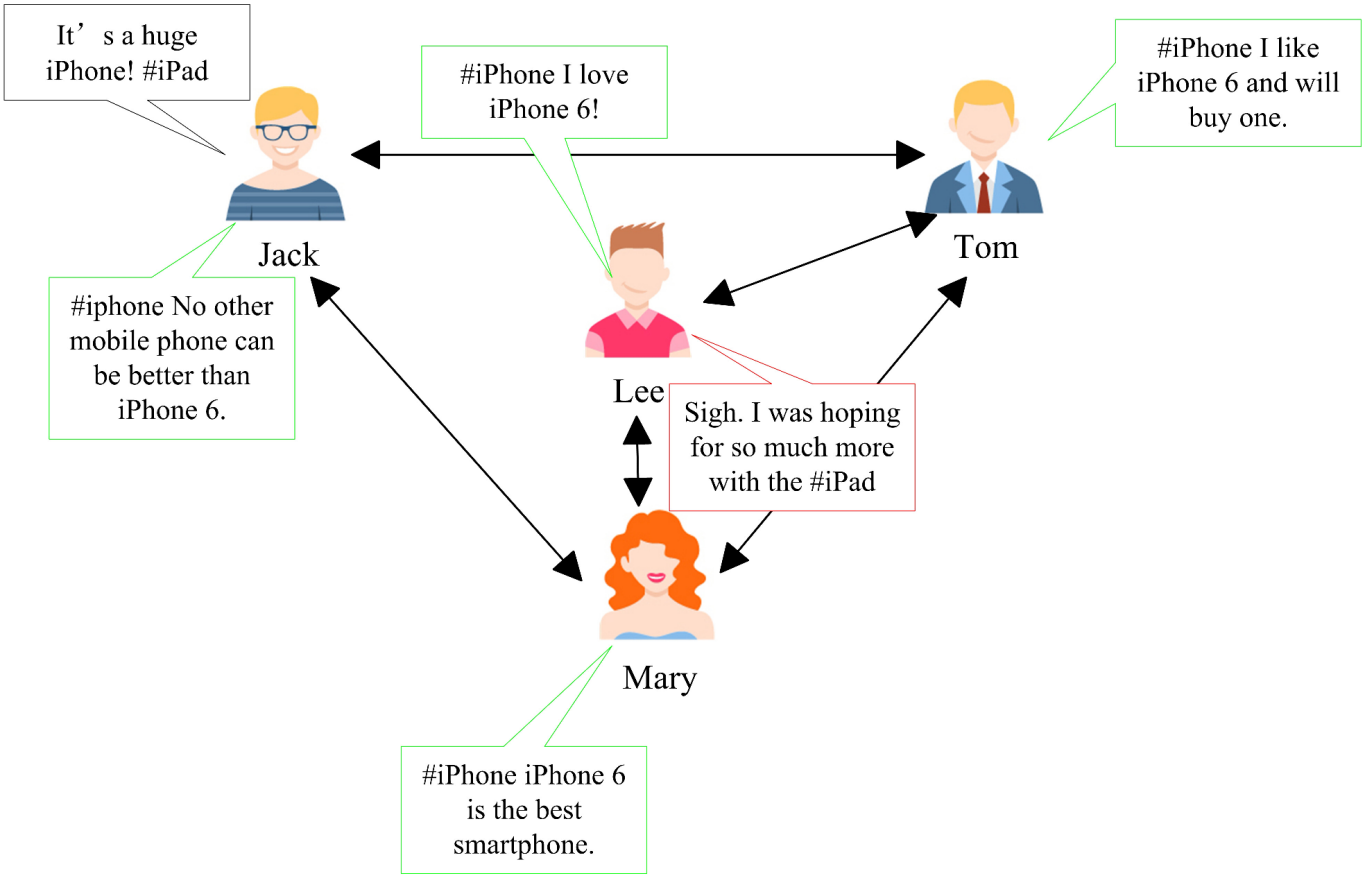
An example. Green dialog boxes represent the corresponding texts are positive, while red dialog boxes represent the corresponding texts are negative.

Studying the influence of other metadata beyond texts (called social context) on microblog sentiment analysis has recently attracted much attention of many researchers, for example, introducing user direct relations to microblog sentiment analysis models [9, 10]. There are two basic sociological theories: sentiment consistency [11] and emotional contagion [12] to support these methods. As an aspect of social context, sentiment consistency, which is called user context, indicates that microblogs posted by the same person tend to have the same sentiment label. Emotional contagion implies that similar people tend to have the same opinion, and it is usually called friends context, which is also an aspect of social context. Although there are already works [9, 10] which exploit social context for sentiment analysis in microblogging, they only take the effects of user direct relationships on sentiment analysis into account, ignoring the impact of user indirect relationships. However, connections in a social network are heterogeneous in nature [13–15], so it is not enough to analyze microblog sentiment by only using user direct relationships. Here is an example. In Fig 1, a green dialog box represents that the sentiment of its corresponding text is positive while the sentiment of the text in a red dialog box is negative. The text in the black dialog box represents the one needed to be classified. There are no direct connections between Jack and Lee, but they have two common friends (Mary and Tom). All users have a positive opinion for iPhone 6. Jack posts a tweet about iPad: “It’s a huge iPhone!” which is a negative comment towards iPad. However, it is difficult to recognize its polarity for a machine from its literal meaning. Besides, if we use direct relationships between users in this graph to assist sentiment analysis, we still can not classify this text into the right class as Lee’s friends (Mary and Tom) have no comments on iPad, which results in a classification error.

Recently, indirect relationships between users have been applied into recommendation systems [16, 17]. The basic idea of these works is that similar users have the same preferences or behavior habits. However, there is little literature that studies the usefulness of indirect relationships in sentiment analysis. At the same time, with the development of sociological theory, homophily [18, 19] has received much more attention [17, 20]. It is the principle that a contact between similar people occurs at a higher rate than among dissimilar people [20], which has a great influence on the formation of friendships. As a result of homophily, the information such as culture and behavior that flows through the network tends to be localized. In addition, [21] has found some evidence of both positive and negative sentiment homophily in social networks.

Inspired by these works, we propose our own method: using indirect relations, in particular, user structure similarity to analyze microblog sentiment. Our method is based on an assumption: opinions of similar users should be similar, and we experimentally verify this assumption. First, we find similar users through common friend relationships and establish a similarity context matrix. It is a common practice of finding similar users through common friends [17, 20, 22, 23] and similarity breeds new connection [19]. Further, two users who may have a new connection between them may share the same opinion [20]. The essence of our method is to look for potential user relationships that may be friends, and then take them into account in the sentiment analysis model. Second, topic factors are introduced and a topic context matrix is established. The phenomenon of homophily is more significant on the same topic [24], and in turn, the topic context can better exploit the theory of homophily. Finally, the structure similarity context and topic context are combined into a graph model, and the Laplacian matrix of this graph is used to analyze microblog sentiment. Go back to the example given in Fig 1 again. Jack and Lee have two common friends. According to our assumption, they have a certain probability of becoming friends, so they may share the same sentiment with a certain probability. Therefore, due to Lee’s negative comment on #iPad, Jack may also have a negative comment on #iPad, then the accuracy of sentiment analysis is guaranteed.

The main contributions of this paper include:

1. Proposing a method using structure similarity to model homophily in social networks.
2. Introducing structure similarity into social context of microblogs as a substitute to user direct relations.
3. Introducing topic context to model the semantic relations between microblogs.
4. Proposing a novel microblog sentiment analysis model which incorporates user context, structure similarity context, topic context and text information.
5. Evaluating the proposed model extensively using real-world datasets to understand the working of the proposed model.

The remainder of this paper is organized as follows. In Section 2, several related works are introduced. In Section 3, we define the problem we study and propose our model. In Section 4, the experimental results are presented. In Section 5, we conclude the whole paper.

## Related work

In this section, we review some related works about sentiment analysis and microblog sentiment analysis.

### Sentiment analysis

Existing approaches to sentiment analysis fall into two main categories: lexicon-based methods and machine learning methods. Lexicon-based methods [25–32] usually utilize lexicon such as SentiWordNet [33], SenticNet [34] to tag words occurring in the sentence into positive and negative labels, then the sentiment of the whole document is judged by summarizing the tagged words. Lexicon-based methods are unsupervised which don’t need datasets with polarity labels. However, these methods rely on lexicons too much and are domain-related for the sentiment polarity of words varies from domain to domain.

Machine learning methods regard sentiment analysis as a text classification problem [35–42]. In these methods, features such as unigrams, bigrams, word embeddings are extracted from the text firstly and then features are fed to classification models such as SVM, NB and deep neural networks (CNNs, RNNs) and so on. Machine learning methods are supervised and usually need lots of training data with polarity labels. The accuracy of sentiment classification is related to the size of training data.

### Microblog sentiment analysis

Microblog sentiment analysis has become a hot research topic in these years [10, 43, 44]. Because microblogs are short and noisy, many methods are proposed to solve this problem. [45] used emoticons as features to analyze the sentiment of tweets. In [46], generalized emoticons, repeated punctuations, and repeated words were used to build a co-occurrence graph by label propagation algorithm and the co-occurrence graph was used to identify the sentiment polarities of tweets. [47] built a sentiment lexicon using the relations between words and emoticons, then they used the lexicon to extract sentiment features and analyze microblogs. All these methods mentioned above utilize text information only and ignore the extra information provided by the microblog media.

In recent years, there are more and more research works about how to utilize user information to analyze sentiment. [10] proposed a method using user follow relations and ‘@’ information to identify the sentiment of users on Twitter. [48] took sentiment analysis of users to a specific topic as a problem of collaborative filtering, relations between users were applied to predict sentiment of users. Similarly, [49] also exploited user relations graph. The classification results of the maximum entropy model were used as labels and then the authors implemented label propagation algorithm to identify sentiment. These works are user-level or user-topic level sentiment classification methods, while our methods are microblog-level. In [9], Hu et al. proposed a framework named SANT (a **S**ociological **A**pproach to handling **N**oisy and short **T**exts) combining social context to classify sentiment of microblogs. On the basis of [9], [50] added content similarity to the framework of SANT and proposed a semi-supervised method to identify sentiment of tweets. [51] argued the framework proposed by [9] was a purely content-based approach so they proposed a **S**tructured **M**icroblog **S**entiment **C**lassification (SMSC) framework which used social context at the prediction stage. There are also works which introduced user relations into microblog retrieval [52, 53]. However, all these methods employ direct user relations and ignore user similarity. Base on the observation in Section 1, two users who have common friends may share the same sentiment with each other, which means using direct user relations only are not enough for sentiment analysis.

## Model

### Datasets

In this paper, our experiments are conducted on two Twitter sentiment analysis benchmark datasets: HCR and OMD. Many proposed works use these two datasets to evaluate the performance of using social relations for sentiment analysis. These two datasets include raw texts and sentiment labels which are labeled manually.

HCR: This dataset is collected by [49]. It includes tweets about health care reform of America in March 2010. It has three parts: a training set, a development set, and a test set. There are five kinds of labels in the dataset: positive, negative, neutral, irrelevant and unsure and this corpus was manually annotated by the authors. In this paper, we only use tweets with positive and negative labels. We use the complete follower graph built by [54] in 2009 to construct the user relations of HCR and take the graph as undirected. The dataset has 9 different topics, i.e. health care reform, Obama, Republicans, Democrats, conservatives, liberals, Tea Party, Stupak and other. Each microblog corresponds to one of these targets.

OMD: This dataset is built by [55]. It consists of tweets discussing the US Presidential Debates between Barack Obama and John McCain. This dataset is manually labeled by Amazon Mechanical Turk. Every tweet is tagged by at least three Turkers and its inter-annotator agreement is 0.655 reported in [55], which shows a relatively good agreement between annotators. Four kinds of labels appear in the dataset, they are positive, negative, mixed and irrelevant. We use majority voting to determine the final label of each tweet. The same as HCR, we only use tweets with positive and negative labels. The relation graph is also built by using the follower graph clawed by [54] in 2009. Microblogs in this dataset can be divided into three topics by using keywords, i.e., Obama (containing keyword “Obama” but no “McCain”), McCain (containing keyword “McCain” but no “Obama”), and debate (containing both “Obama” and “McCain” or none of them).

In this paper, we reserve users who have friends and delete those microblogs whose author has no friends. The information about the two datasets is shown in Table 1.

**Table 1:** Statistics of datasets.

Emoticon	HCR	OMD
# of Tweets	1434	1184
# of users	806	636
# positive Tweets	387	475
Average Tweets per User	1.78	1.86
Average Friends per User	14.95	5.54

### Notation

In this paper, uppercase letters like *B* are used to denote matrices, lowercase bold letters like **x** denote vectors. Lowercase letters like *a* denote scalar values. We use *B*_*i*∗_ to denote the *i*-th row of matrix *B* and *B*_∗*j*_ to denote the *j*-th column of matrix *B*. The entry at the *i*-th row and *j*-th column is denoted as *B*_*ij*_. *B*^*T*^ is used as the transposition of matrix *B*. ‖*B*‖_*F*_ denotes the Frobenius norm of *B*. *tr*(.) is the trace of a matrix.

The goal of this paper is by using the training set feature matrix *X* ∈ *R*^*n*×*m*^ (where *n* represents the number of microblogs in training set, *m* represents the number of features.) and label matrix *Y* ∈ *R*^*n*×*c*^ (where *c* is the number of sentiment polarities) to construct a classifier *W* ∈ *R*^*m*×*c*^, and then classifier *W* ∈ *R*^*m*×*c*^ was used to predict an unseen microblogs **x**. *Y* represents ground truth labels of microblogs. We use $\hat{Y}=XW\in R^{n\times c}$ to represent the fitted value of the ground truth label matrix *Y*. In this paper, we only consider binary classification of sentiments, that is, *c* = 2. Therefore, if a microblog is positive, then its ground truth label is *Y*_*i*∗_ = [+1 −1]. And if the sentiment of a microblog is negative, then its label is *Y*_*i*∗_ = [−1 +1].

Given an undirected graph *G* = (*V*, *E*), *A* represents its adjacency matrix, *L* = *D* − *A* represents the Laplacian matrix of *G* [56], where *D* is diagonal matrix and *D*_*ii*_ indicates the degree of the *i*-th vertex.

To classify an unseen microblog, we use the prediction function in Eq (1). Variables and their meanings are shown in Table 2.

$$\begin{array}{c} g\left(x\right)=\{\begin{array}{ccc} +1 & if & xW_{*1}>xW_{*2}\\ -1 & if & xW_{*1}<xW_{*2}\\ +1 & or & -1\;randomly\;if\;xW_{*1}=xW_{*2} \end{array} \end{array}$$ (1)

**Table 2:** Meaning of variables.

Variables	Meaning	Type
uppercase letter like *B*	matrix	–
lowercase letters like *b*	scalar	–
lowercase bold letters like **b**	vector	–
*B*_∗*i*_	the *i*-th column of matrix *B*	
*B*_*i*∗_	the *i*-th row of matrix *B*	
*X*	feature matrix	*R*^*n*×*m*^
*Y*	ground truth label matrix	*R*^*n*×*c*^
$\hat{Y}$	fitted sentiment label matrix	*R*^*n*×*c*^
*n*	number of features	integer
*m*	number of training set	integer
*c*	number of sentiment classification	integer
*t*	number of topics	integer
*W*	classifier	*R*^*m*×*c*^
**x**	feature vector of a microblog	*R*^*m*^
*U*	user-microblog matrix	*R*^*d*×*n*^
*d*	number of users	integer
*S*	structure similarity matrix	*R*^*d*×*d*^
*T*	microblog-topic matrix	*R*^*n*×*t*^
*M*	microblog-microblog topic matrix	*R*^*n*×*n*^
*A*	microblog-microblog relation matrix	*R*^*n*×*n*^
*D*	diagonal matrix	*R*^*n*×*n*^
*L*	Laplacian matrix	*R*^*n*×*n*^
F	user-user direct relation matrix	*R*^*d*×*d*^
*G*	graph	
*E*	edges	
*V*	nodes	

### Modeling microblog content

The popular method Least Squares is applied to fit the classification model for text information. In terms of multiclass classification tasks, the Least Squares aims to learn *c* classifiers by solving the Eq (2) optimization problem:

$$\begin{array}{c} \min_{W}\frac{1}{2}{∥XW-Y∥}_{F}^{2} \end{array}$$ (2)

Unlike traditional text information, microblogs are short and have many noises which lead to a sparse matrix of unigrams. To handle this problem, we use sparse regularization *L*_1_ norm to seek a sparse reconstruction of the feature space. To minimize the *L*_1_ norm based linear reconstruction error can implement feature selection automatically and get a sparse representation of texts [57]. Thus, we add *L*_1_ norm in our model to get a more robust model (see Eq (3)).

$$\begin{array}{c} \min_{W}f\left(W;X,Y\right)=\min_{W}\frac{1}{2}{∥XW-Y∥}_{F}^{2}+\beta {∥W∥}_{1} \end{array}$$ (3)

where *β* is the weight of regularization.

### Context besides text

In this section, we will introduce the different contexts used in this paper and integrate them into the final model.

#### Topic context

In this section, we introduce the topic context. Hashtags are a type of mechanisms provided by microblogging services, by which users can insert topic information into microblogs conveniently. For example, in a tweet (Twitter microblog message), the symbol # is used to tag topics in a tweet. A tweet “I love #iPhone6” indicates that this tweet is about “iPhone6”. Users post various microblogs towards different topics in social media as a way to express themselves. Although different users may have different opinions towards the same topic and a user may hold different opinions towards different topics, the opinions of a same person on the same topic usually consistent with each other. In addition, similar users tend to hold similar opinions towards the same topic. Topic context is used to indicate whether two microblog messages are related to the same topic. It is important to introduce topic information into microblog sentiment analysis as it models the semantic connections between microblogs. It is noted that we use topics not text similarity to model this semantic relation. This is because the data representation is very sparse in microblogging platform, and if we use text similarity the values of semantic similarity between microblogs will be very small which cannot model the semantic relation efficiently. We can get a microblog-microblog matrix *M* towards topics using Eq (4), where *T* ∈ *R*^*n*×*t*^ is the microblog-topic matrix and *T*_*ij*_ = 1 if and only if the *i*-th microblog is about the *j*-th topic.

$$\begin{array}{c} M=T\times T^{T} \end{array}$$ (4)

*M*_*ij*_ = 1 if and only if microblog *p*_*i*_ and *p*_*j*_ are about the same topic. The diagonal elements of *M* are set to zeros.

#### User context

User context is based on a sociological theory called sentiment consistency. Sentiment consistency suggests that the sentiments of two microblogs posted by the same user have a higher probability to be the same than those of two randomly selected microblogs, which has been verified in [9] and [10]. *A*_*sc*_ ∈ *R*^*n*×*n*^ represents microblog-microblog matrix for sentiment consistency. We can use Eq (5) to calculate *A*_*sc*_. *U* ∈ *R*^*d*×*n*^ is a user-microblog matrix where *U*_*ij*_ = 1 if the *i*-th user posts the *j*-th microblog and *d* is the number of users.

$$\begin{array}{c} A_{sc}=U^{T}\times U \end{array}$$ (5)

*A*_*sc*__*ij*_ = 1 if and only if microblog *p*_*i*_ and *p*_*j*_ are posted by the same user.

#### Structure similarity context

This part is also based on a basic theory of sociology: emotional contagion, which discloses that the sentiments of two microblogs posted by similar users have a higher probability than those of two randomly selected microblogs. In previous work, if two microblogs are posted by two users connected with follower/friend relationships, a model is built to make the sentiments of these two microblogs as close as possible. It is called friends context, represented by *A*_*ec*_ = *U*^*T*^ × *F* × *U*, where *F* ∈ *R*^*d*×*d*^ represents a user-user matrix and *F*_*ij*_ = 1 if there exists a following/followee relation between the *i*-th user and the *j*-th user. However, previous works only use direct relations between users and ignore common friendships between users. As discussed in Section 1, a user may also share the same opinion with the user who is a friend of his friends, which is an expression of homophily. Therefore, in this part, we use structure similarity which takes common friendships into consideration to model the emotional contagion theory. Common friendships often induce new friendships [58]. In real life, if B and C have a common friend A, the probability of becoming friends between them increases. This principle is called “Triadic Closure” [59, 60]. One of the reasons for triadic closure is the fact that both B and C are friends of A (as they all know it) provides them with the basic trust that is lacked among strangers during the formation of friendships. The second reason is based on A’s incentive: it can decrease the latent stress of A in two separate relationships to bring B and C together.

**Fig 2:**
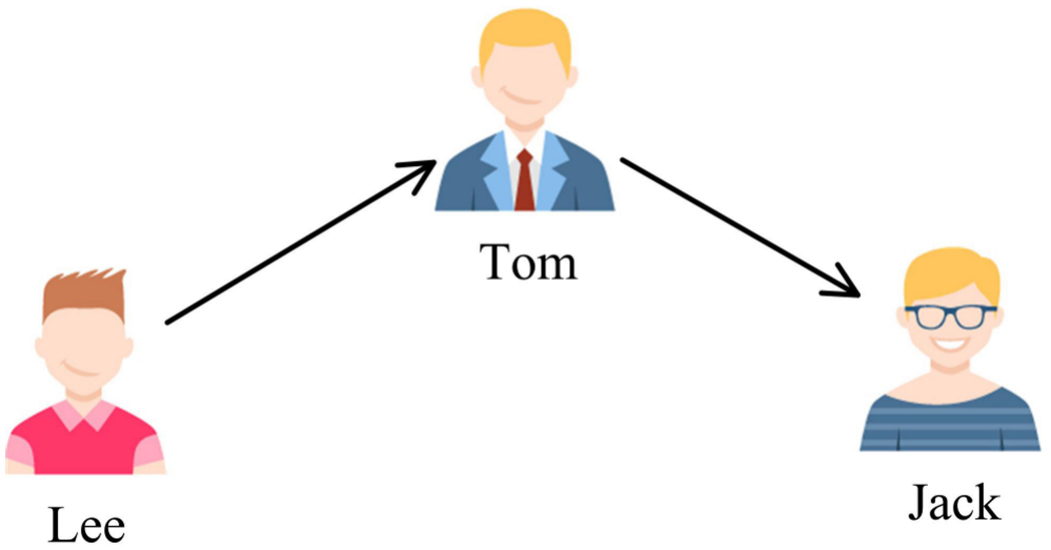
Different relation types in Twitter: The first case.

**Fig 3:**
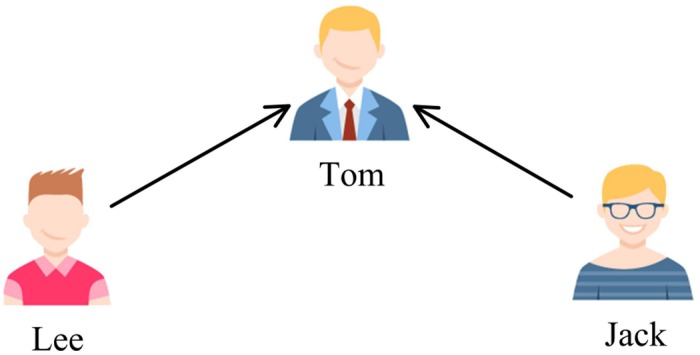
Different relation types in Twitter: The second case.

**Fig 4:**
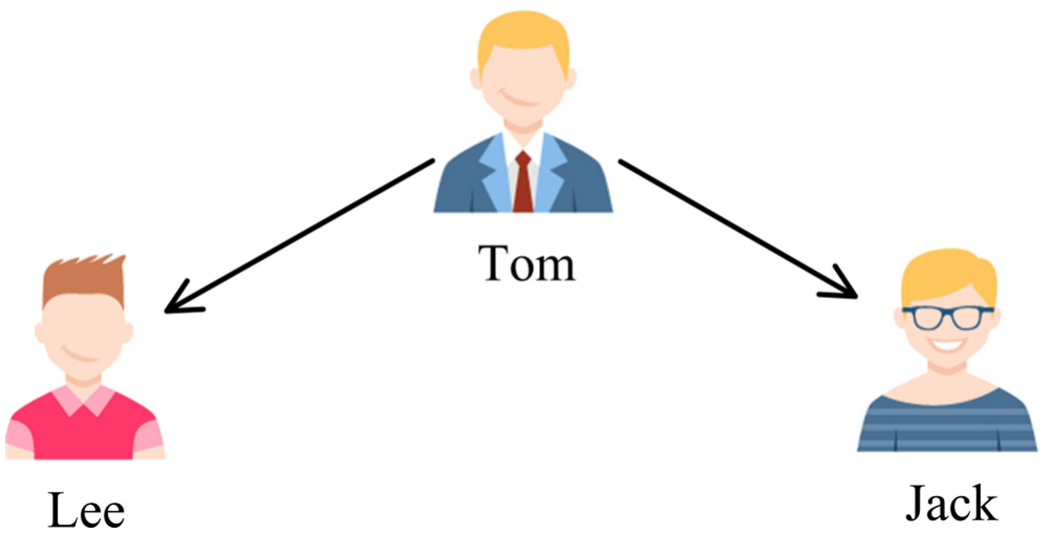
Different relation types in Twitter: The third case.

There are three cases that three users are connected by two following relations in Twitter. This is shown in Figs 2, 3 and 4, where the user pointed by the arrow is a followee and the user at the other end of the arrow is a follower. The first case (Fig 2) represents the process of the flow of information, an opinion may flow from Jack to Lee through Tom. The second one (Fig 3) describes a situation that two users share a common followee. Sharing a common followee emphasizes the establishment of friend-of-friend relationships, which means that the more common followees between two users, the easier it is to build a following relation between them. The third one (Fig 4) demonstrates the situation that two users have a common follower. This case reflects the similarity of two users’ image and attractiveness. No matter what the case is, all the three cases are an expression of user similarity, which implies the possibility of the formation of a friendship between the two unconnected users. For this reason, we can take the follower graph as undirected.

Given two users *u*_*i*_ and *u*_*j*_, their structure similarity can be calculated by Eq (6).

$$\begin{array}{c} S_{ij}=Sim\left(u_{i},u_{j}\right)=|N_{u_{i}}⋂N_{u_{j}}| \end{array}$$ (6)

**Fig 5:**
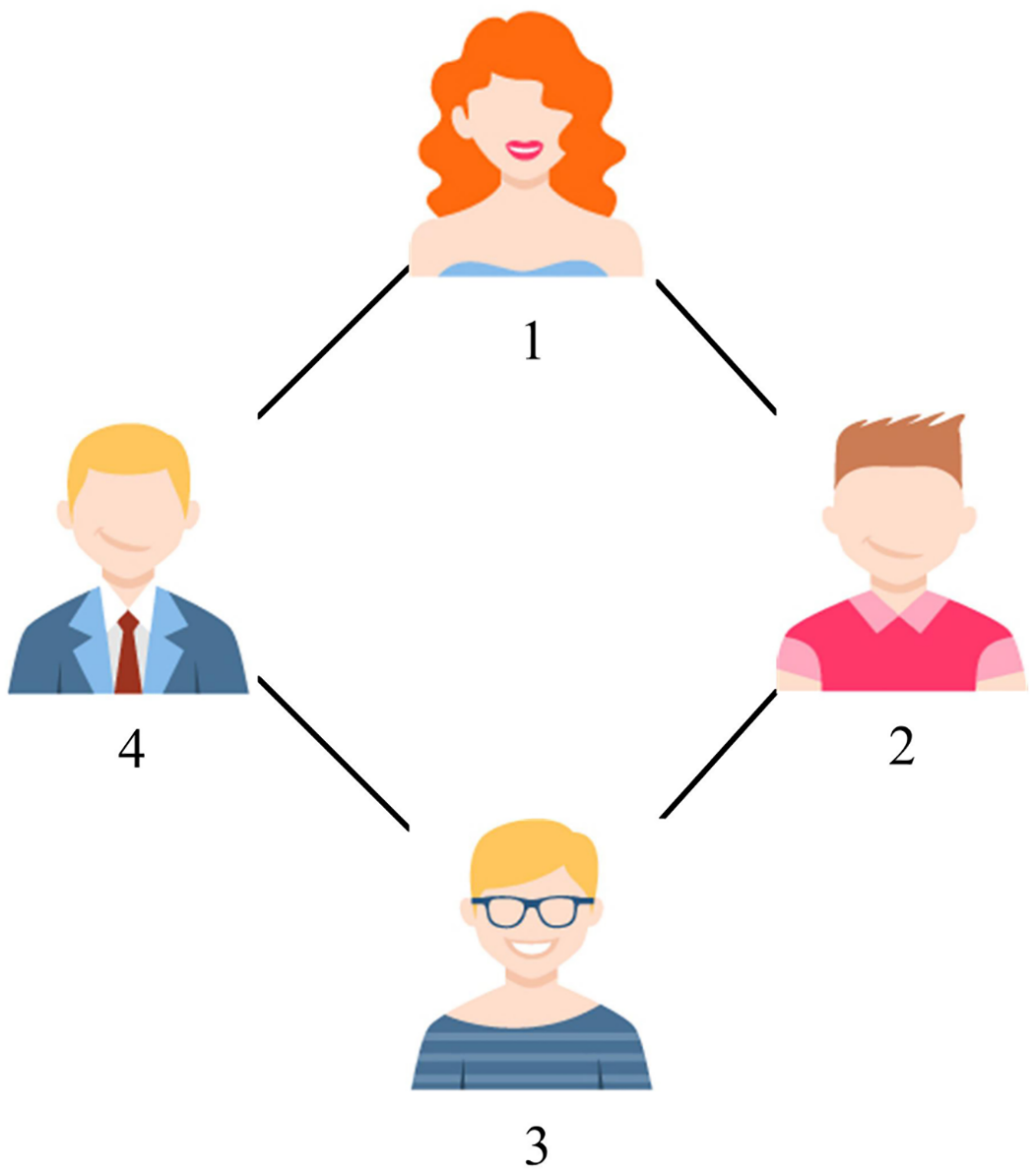
Example of similarity (a).

**Fig 6:**
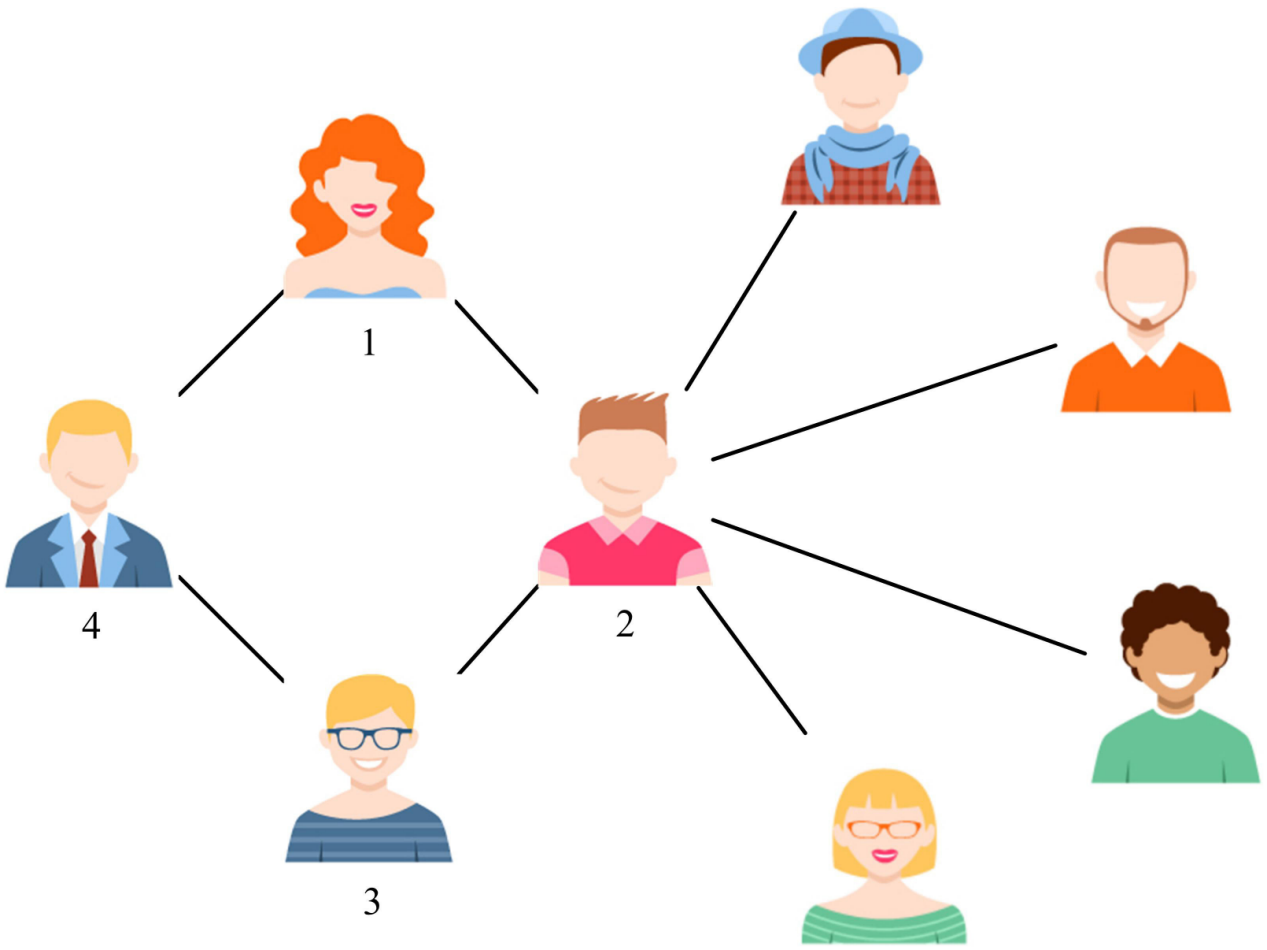
Example of similarity (b).

The structure similarity is measured by two users’ common friends. *N*_*u*_*i*__ represents neighbours of user *u*_*i*_. |*N*_*u*_*i*__ ⋂ *N*_*u*_*j*__| represents the number of *u*_*i*_ and *u*_*j*_’s common friends. However, considering a condition, shown in Fig 5, user 2 and user 4 have two common friends 1 and 3. Compared with Fig 5, in Fig 6, user 2 has many more friends. If we use Eq (6) to compute the structure similarity between user 2 and user 4 of Fig 6, we will get the same similarity value as that in Fig 5. To handle this problem, we use Eq (7) in which all neighbors of two users are taken into consideration to compute the structure similarity.

$$\begin{array}{c} S_{ij}=Sim\left(u_{i},u_{j}\right)=\left\{ \begin{array}{c} \frac{|N_{u_{i}}⋂N_{u_{j}}|}{|N_{u_{i}}⋃N_{u_{j}}|}\;for\;two\;unconnected\;users.\\ \frac{|N_{u_{i}}⋂N_{u_{j}}|}{|N_{u_{i}}⋃N_{u_{j}}|}+1\;for\;two\;connected\;users. \end{array}\right. \end{array}$$ (7)

where *N*_*u*_*i*__ ⋃ *N*_*u*_*j*__ represents the union set of friends of both user *u*_*i*_ and *u*_*j*_ and |*N*_*u*_*i*__ ⋃ *N*_*u*_*j*__| represents the number of users in the union set.

After getting user structure similarity matrix *S*, the emotional contagion matrix *A*_*ec*_ can be computed by Eq (8).

$$\begin{array}{c} A_{ec}=U^{T}\times S\times U \end{array}$$ (8)

### Incorporating structure similarity context

In this section, we combine the three kinds of contexts into our framework. *A*_1_ ∈ *R*^*n*×*n*^ is used to represent the combination of user context and sturcture similarity context. It can be calculated by Eq (9). *A*_2_ ∈ *R*^*n*×*n*^ represents the combination of user context, structure similarity context, and topic context. Eq (10) can be used to compute *A*_2_. We set *θ* = 1.

$$\begin{array}{c} A_{1}=A_{sc}+\theta *A_{ec} \end{array}$$ (9)

$$\begin{array}{c} A_{2}=\left(A_{sc}+\theta *A_{ec}\right)\circ M \end{array}$$ (10)

where ∘ represents Hadamard product.

We use the SANT framework proposed by [9]. Based on sentiment consistency and emotional contagion, to integrate sentiment relations between microblogs in sentiment classification, the basic idea is to make two microblogs as close as possible if they are posted by the same user or two users are very similar with each other. In this situation, we can solve the problem by minimizing Eq (11).

$$\begin{array}{ccc} & & \min_{W}\frac{1}{2}\sum_{i=1}^{n}\sum_{j=1}^{n}A_{ij}{∥{\hat{Y}}_{i*}-{\hat{Y}}_{j*}∥}^{2}\\ & & \;=\min_{W}\sum_{k=1}^{c}{\hat{Y}}_{*k}^{T}\left(D-A\right){\hat{Y}}_{*k}\\ & & \;=\min_{W}tr\left(W^{T}X^{T}LXW\right) \end{array}$$ (11)

If we only use user context and structure similarity context, *A* = *A*_1_. If topic context is used, *A* = *A*_2_. So the final model which combines text information and social context can be represented by Eq (12).

$$\begin{array}{c} f\left(W;X,Y\right)=\min_{W}\frac{1}{2}{∥XW-Y∥}_{F}^{2}+\frac{\alpha }{2}tr\left(W^{T}X^{T}LXW\right)+\beta {∥W∥}_{1} \end{array}$$ (12)

where *α* is the weight of social context in the model, *β* is the weight of regularization.

### Learning

Motivated by [61], we solve the non-smooth optimization problem in Eq (12) by optimizing its equivalent smooth convex reformulations. Firstly, Eq (12) can be reformulated as Eq (13) as a constrained smooth convex optimization problem.

$$\begin{array}{ccc} & & \min_{W\in Z}L\left(W;X,Y\right)=\frac{1}{2}{∥XW-Y∥}_{F}^{2}+\frac{\alpha }{2}tr\left(W^{T}X^{T}LXW\right),\\ & & where\;Z=\{W|{∥W∥}_{1}\leq z\} \end{array}$$ (13)

*L*(*W*;*X*, *Y*) is the differentiable part and *Z* is the non-differentiable part. *z* ≥ 0 is the radius of the *L*_1_-ball, and there is a one-to-one correspondence between *β* and *z*.

The smooth part of the optimization problem can be reformulated equivalently as a proximal regularization [62] of the linearized function *L*(*W*;*X*, *Y*) at *W*_*t*_, which is formally defined as:

$$\begin{array}{ccc} & & W_{t+1}=\arg\min_{W}G_{\lambda_{t},W_{t}}\left(W\right)\\ & & where\;G_{\lambda_{t},W_{t}}\left(W\right)=L\left(W_{t};X,Y\right)+\\ & & <▽L\left(W_{t};X,Y\right),W-W_{t}>+\frac{\lambda_{t}}{2}{∥W-W_{t}∥}_{F}^{2} \end{array}$$ (14)

where λ_*t*_ is the step size in the *t*-th iteration. In this paper, the gradient of *L*(*W*;*X*, *Y*) with respect to *W* can be computed using Eq (15).

$$\begin{array}{c} ▽L\left(W;X,Y\right)=X^{T}\left(XW-Y\right)+\alpha X^{T}LXW \end{array}$$ (15)

When considering the constraints *Z* in Eq (13), and given *β*, the (*t*+1)-th *W* can be computed by Eq (16).

$$\begin{array}{c} {\left(W_{t+1}\right)}_{j*}=\left\{ \begin{array}{c} \left(1-\frac{\beta }{\lambda_{t}∥{\left(U_{t}\right)}_{j*}∥}\right){\left(U_{t}\right)}_{j*},if∥{\left(U_{t}\right)}_{j*}∥⩾\frac{\beta }{\lambda_{t}}\\ 0,otherwise \end{array}\right. \end{array}$$ (16)

where $U_{t}=W_{t}-\frac{1}{\lambda_{t}}▽L\left(W_{t};X,Y\right)$. As discussed in [61], to achieve the optimal convergence, we can further accelerate our constrained smooth convex optimization problem. In particular, two sequences *W*_*t*_ and *V*_*t*_ are used in this accelerated algorithm. *W*_*t*_ is the sequence of approximate solutions, and *V*_*t*_, an affine combination of *W*_*t*_ and *W*_*t*−1_, is the sequence of search points. *V*_*t*_ can be computed by Eq (17).

$$\begin{array}{c} V_{t}=W_{t}+\gamma_{t}\left(W_{t}-W_{t-1}\right) \end{array}$$ (17)

Where *γ*_*t*_ is the combination coefficient. The approximate solution *W*_*t*+1_ is computed as a “gradient” step of *V*_*t*_ through *G*_λ_*t*_,*V*_*t*__. We use Nesterov’s method [63] to solve the optimization problem. The details are shown in Algorithm 1 in which *η*_*t*_ is set according to [61].

**Algorithm 1 SASS**: Sentiment analysis using structure similarity

**Input:** *X*, *Y*, *L*, *α*, *β*

**Output:** *W*

1. Initialize *W*_0_ by random

2. Set *η*_0_ = 0, *η*_1_ = 1, *W*_1_ = *W*_0_, *t* = 1

3. **while** not convergent **do**

4.   Compute $V_{t}=W_{t}+\frac{\eta_{t-1}-1}{\eta_{t}}\left(W_{t}-W_{t-1}\right)$

5.   Compute ▽*L*(*W*_*t*_;*X*, *Y*)

6.   **while** True **do**

7.     Compute $U_{t}=V_{t}-\frac{1}{\lambda_{t}}▽L\left(W_{t};X,Y\right)$

8.     Compute *W*_*t*+1_ according to Eq (16)

9.     **if** *L*(*W*_*t*+1_, *X*, *Y*)≤*G*_λ_*t*_, *V*_*t*__(*W*_*t*+1_) **then**

10.       Set λ_*t*+1_ = λ_*t*_

11.       Break

12.     **end if**

13.     Set λ_*t*_ = 2 × λ_*t*_

14.   **end while**

15.   **if** *t* > *MaxIter* **then**

16.     Return *W*_*t*+1_

17.   **end if**

18.   Set $\eta_{t+1}=\frac{1+\sqrt{1+4\eta_{t}}}{2}$

19.   Set *t* = *t*+1

20. **end while**

## Experiments

In this section, we present empirical evaluation results to assess the effectiveness of our proposed framework. In particular, we evaluate the proposed method on the two datasets introduced in Section 3. Impacts brought by different contexts and parameters are further discussed.

### Correlation between structure similarity and sentiment

The positive relation between friends context and microblogs sentiment labels are verified in [9] and [10]. In this paper, we also engage in a statistical study of the degree to which structure similarity and microblogs sentiment labels correlate. Given the unweighted graph *G* = (*V*, *E*) built on microblog-microblog relations, we compute the ratio of edges whose corresponding nodes have the same sentiment labels to all edges in *E*, denoted by $\frac{\sum_{i=1}^{n}\sum_{j=1}^{n}1\left(Y_{i*}=Y_{j*},e_{ij}\in E\right)}{\sum_{i=1}^{n}\sum_{j=1}^{n}1\left(e_{ij}\in E\right)}$ where **1**(.) is the indicator function. Given an weighted graph *G*, we can also compute this ratio by taking its weights matrix into consiration, so the ratio can be computed by Eq (18). In Eq (18), weights are regarded as a degree to what the two microblogs have the same sentiment label. [50] also use the index *p* to evaluate the correlation between text similarity and sentiment labels.

$$\begin{array}{c} p=\frac{\sum_{i=1}^{n}\sum_{j=1}^{n}1\left(Y_{i*}=Y_{j*},e_{ij}\in E\right)\cdot A_{ij}}{\sum_{i=1}^{n}\sum_{j=1}^{n}1\left(e_{ij}\in E\right)\cdot A_{ij}} \end{array}$$ (18)

where **1**(.) is the indicator function, *A* represents the weights matrix of *G*.

**Fig 7:**
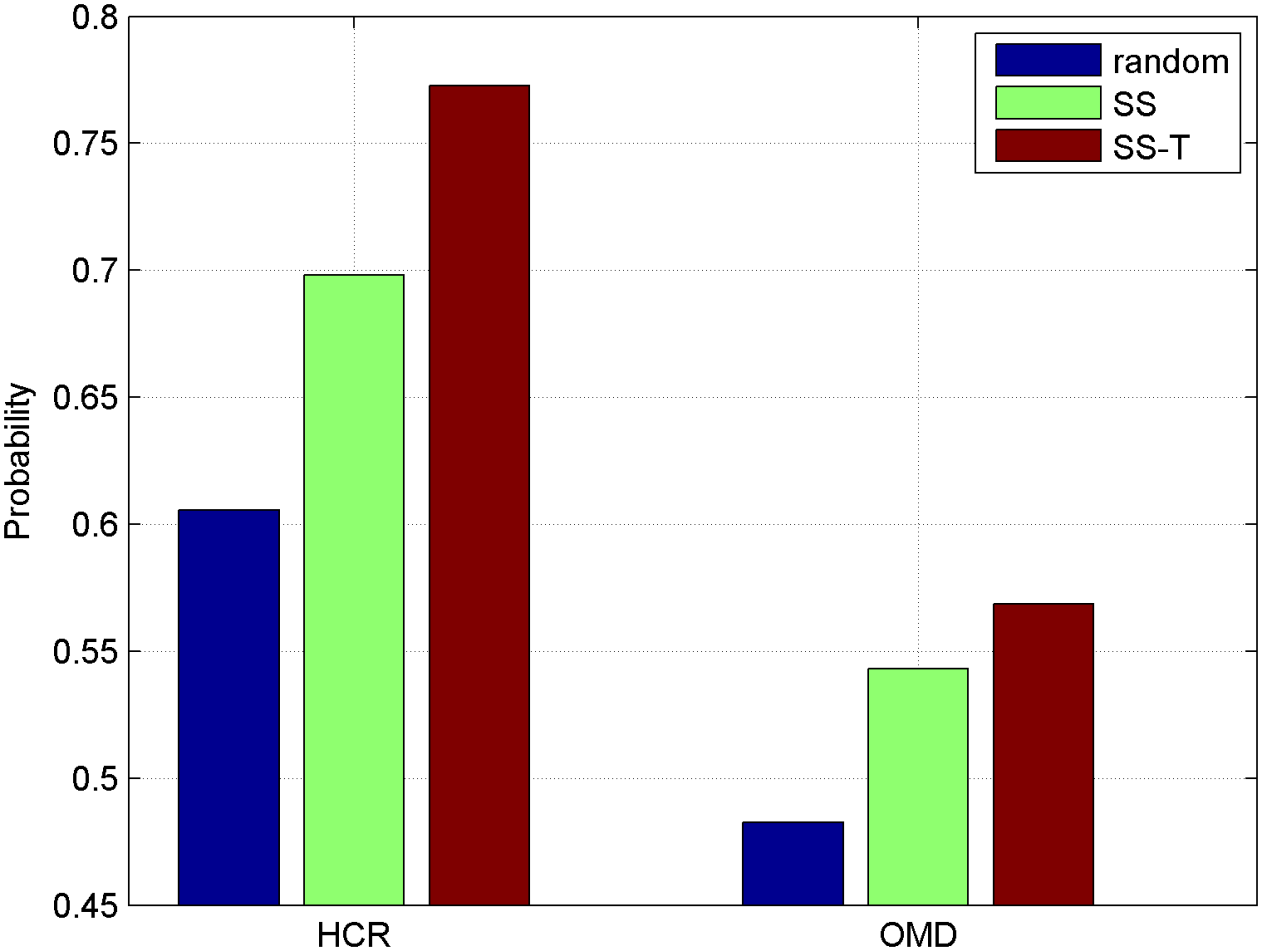
Shared sentiment probability conditioned on structure similarity.

Fig 7 clearly shows the ratio of different methods on both HCR and OMD. SS denotes the microblog-microblog graph constructed by structure similarity, SS-T denotes the microblog-microblog graph built by structure similarity and topic context. We find that the ratio of SS and SS-T is much higher than chance on both HCR and OMD in Fig 7, that is, there is a positive relation between structure similarity and sentiment labels, which paves the way for our next study: how to exploit and model structure similarity into the microblog sentiment analysis system. It is noted that the ratio of SS-T method is higher than SS method. This is because that homophily is more obvious on the same topic which has been verified in [24] and similar people tend to have the same opinion on the same topic. Adding topic context can better exploit the heterogeneous relations between microblogs.

### Usefulness of social context

In this section, we perform experiments to assess the validity of different contexts whether they could improve the accuracy of sentiment classification. We use 90% microblogs for training. “TC” represents the method of only using **t**ext **c**ontext (TC), “UC” represents the method using **u**ser **c**ontext (UC) and texts. Similarly, “SSC” denotes the method combining **s**tructure **s**imilarity **c**ontext (SSC) and texts, “FC” is the method using **f**riends **c**ontext (FC) and text information. We use accuracy, which is the proportion of true results (both true positives and true negatives) among the total number of cases examined, as a metric to measure the performance of different algorithms. It can be computed by *accuracy* = (*TP* + *TN*)/(*num*), where *num* represents the number of both positive samples and negative samples in the training set. *TP* and *TN* represent the number of items correctly labeled as belonging to the positive class and the negative class respectively. The result is shown in Table 3.

**Table 3:** Performance of different context.

	TC	FC	UC	SSC
HCR	0.631944	0.784722	0.770833	0.791667
OMD	0.647058	0.781513	0.773109	0.789916

From this table, we can conclude following observations.

1. Using social context can improve the performance of sentiment analysis on both HCR and OMD datasets. The accuracy of the methods using social context is higher than the accuracy of using text only, which validates the usefulness of user context, friends context and structure similarity context. The performance of social context reveals that sentiment consistency and emotional contagion hold true in microblogging platform and this can be an experimental basis for the two theory.
2. User context gets the lower improvement than the other social context. This is mainly due to the fact that the average number of friends is larger than the average number of microblogs one user posted, which lead to a sparser sentiment consistency matrix. For example, according to Table 1, each user in HCR dataset only has 1.78 tweets on average, while the average number of friends is 14.95.
3. It is also noted that the method using structure similarity context gets the best performance among all social context. Structure similarity can get more information than direct relation such as common friends and weights about whose influence are larger on users, which is the reason behind its better performance than others.

### Performance evaluation

In this section, we use random sampling method to test the accuracy of different methods in different size training set. The methods we use in this paper are listed below.

Least Square (LS): Least Square method [64] is a widely used supervised classifier. Its goal is to find *W* which minimize the function $f\left(X\right)=\frac{1}{2}{∥XW-Y∥}_{F}^{2}$.

Lasso: Lasso [64] only use texts to identify sentiment. Comparing with Least Square method, Lasso adds ‖*W*‖_1_ to handle the sparse problem of classifier *W*.

Support Vector Machine (SVM): SVM [45] is a widely used classifier in the fields of text and hypertext categorization, images classification and so on.

Naive Bayes (NB): Like SVM, NB [45] is also a supervised classifier in many fields.

Logistic Regression (LR): LR [45] denotes *L*_2_-norm regularized Logistic Regression, a popular sentiment classification method.

SANT: A method proposed by [9] which combined sentiment consistency and emotional contagion.

SMSC: A method proposed by [51] which use graph information at the prediction stage.

SASS: Our method of Sentiment Analysis based on Structure Similarity, which uses structure similarity and user context to analyze sentiment.

**Fig 8:**
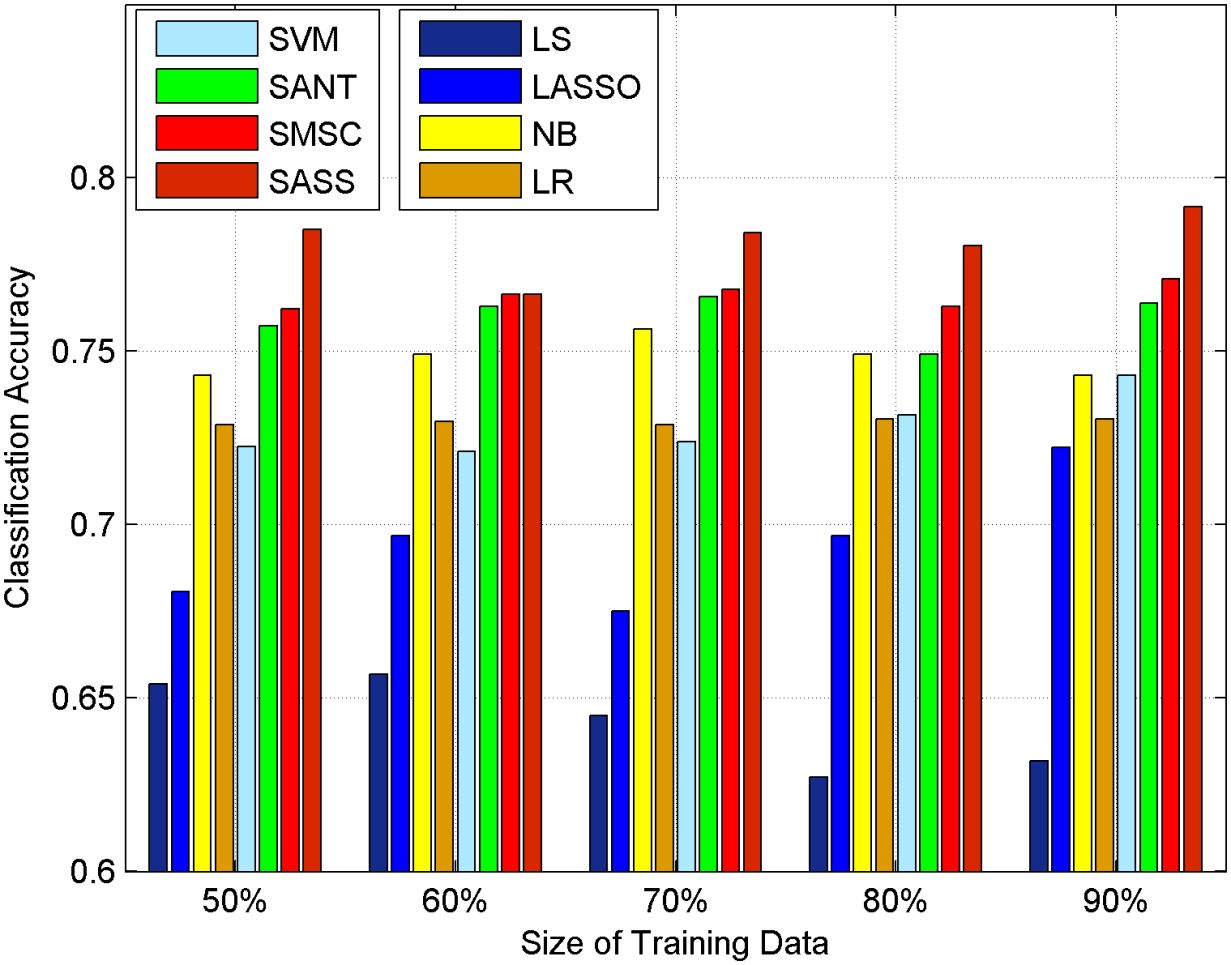
The performance of our method and baseline methods on HCR without topic context.

**Fig 9:**
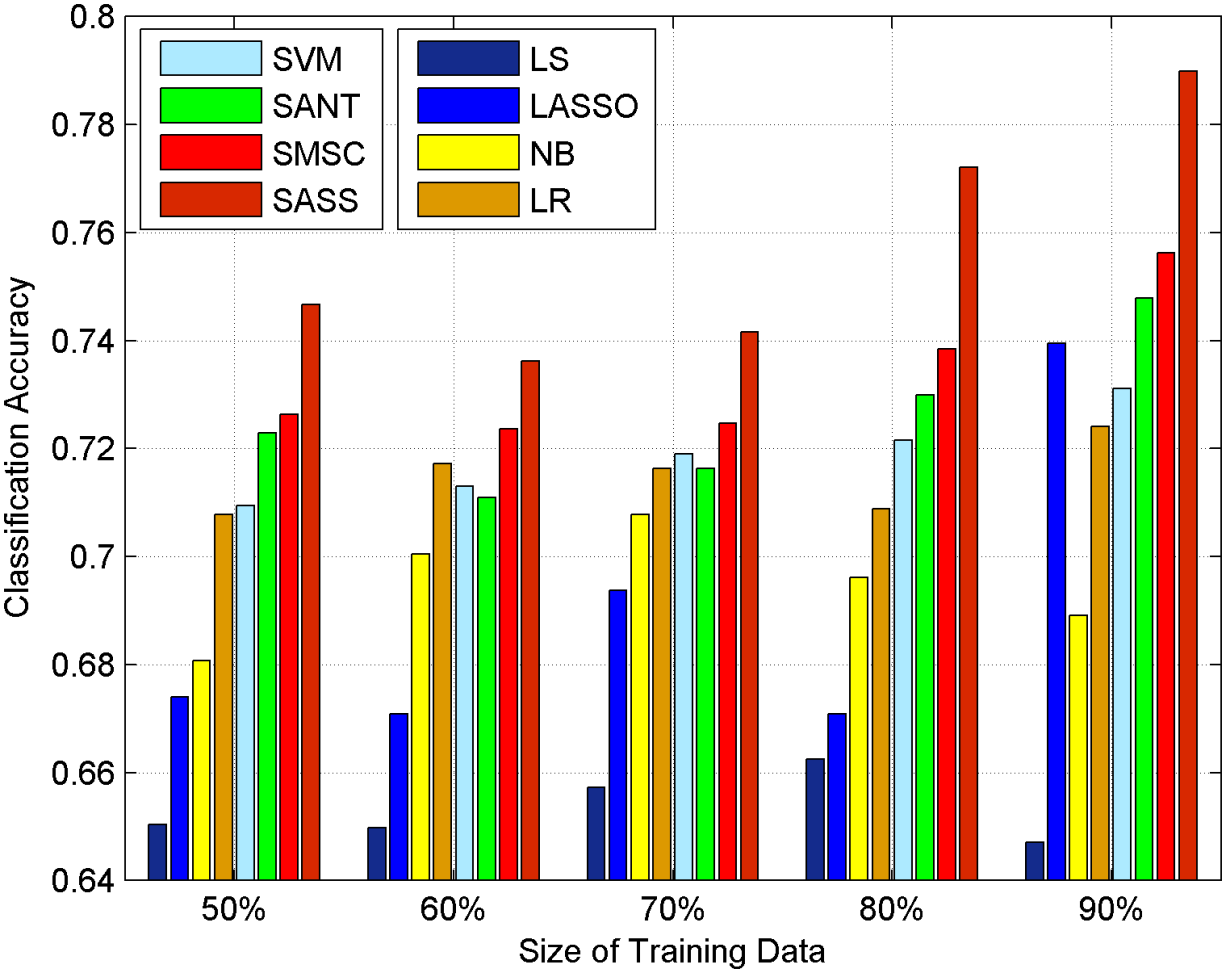
The performance of our method and baseline methods on OMD without topic context.

In our method, there are two import parameters: *α*, *β*. The two parameters are all nonnegative. In this section, we set *α* = 0.0005, *β* = 1 which are tuned by cross-validation. *α* is the parameter that controls the contribution of social context information, *β* is the sparse regularization parameter. The training set and the test set are selected randomly from the original dataset to test our method. p% represents the percentage of the training set, and the rest is used for testing. Experimental results of HCR and OMD are shown in Figs 8 and 9 respectively.

Via comparing the results of different methods, we can draw the following observations.

1. Methods only using texts achieve lower improvement than methods using social context. Two-sample one-tail t tests are conducted and the results show that methods using social context can get improved sentiment classification accuracy with a significance level 0.01. Text information in microblogging platform is very noisy, irony and sarcasm are always used to express the negative feelings of users. Methods such as SVM, LS, LR, and NB cannot handle this situation, while using social context can solve the problem to some extent as they take microblogs which are connected to them into consideration and lead to a better performance.
2. SASS outperforms SANT and SMSC and get the best performance among all methods on both datasets with different sizes of training data consistently and significantly. Compared with the traditional LS method, our proposed method SASS can get the average percentage improvement ($\frac{\left(the\;accuracy\;of\;SASS\;-\;the\;accuracy\;of\;LS\right)}{the\;accuracy\;of\;LS}$) of microblog sentiment analysis accuracy by about [21.61108% and 15.9152%] in HCR and OMD respectively, which outperforms SMSC’s [19.18897% and 12.3313%] and SANT’s [18.19744% and 11.0669%] improvement respectively. This improvement is continuous and significant in both datasets. SANT and SMSC only use user context and friends context. In contrast, our method SASS which uses structure similarity can deeply explore the relations between microblogs. In our method, every microblog has different contributions to the sentiments of other microblogs while in SANT and SMSC all microblogs are regarded to have the same contribution to others. Besides, our method takes potential friendships into consideration. This is the reason that our method can achieve a better performance.
3. Lasso achieves a better performance than LS, this implies using a sparse solution is an effective way to handle noisy microblog texts as it can select features in an automatic way.
4. When there is only 50% data for training, our method still outperforms other methods on OMD and HCR and the performance of SASS is not sensitive to the changes of the size of training data. This demonstrates we can save a lot of cost in labeling, which has its significance to solve the problem of “lack of manually labeled training data”.

### Usefulness of topic context

**Fig 10:**
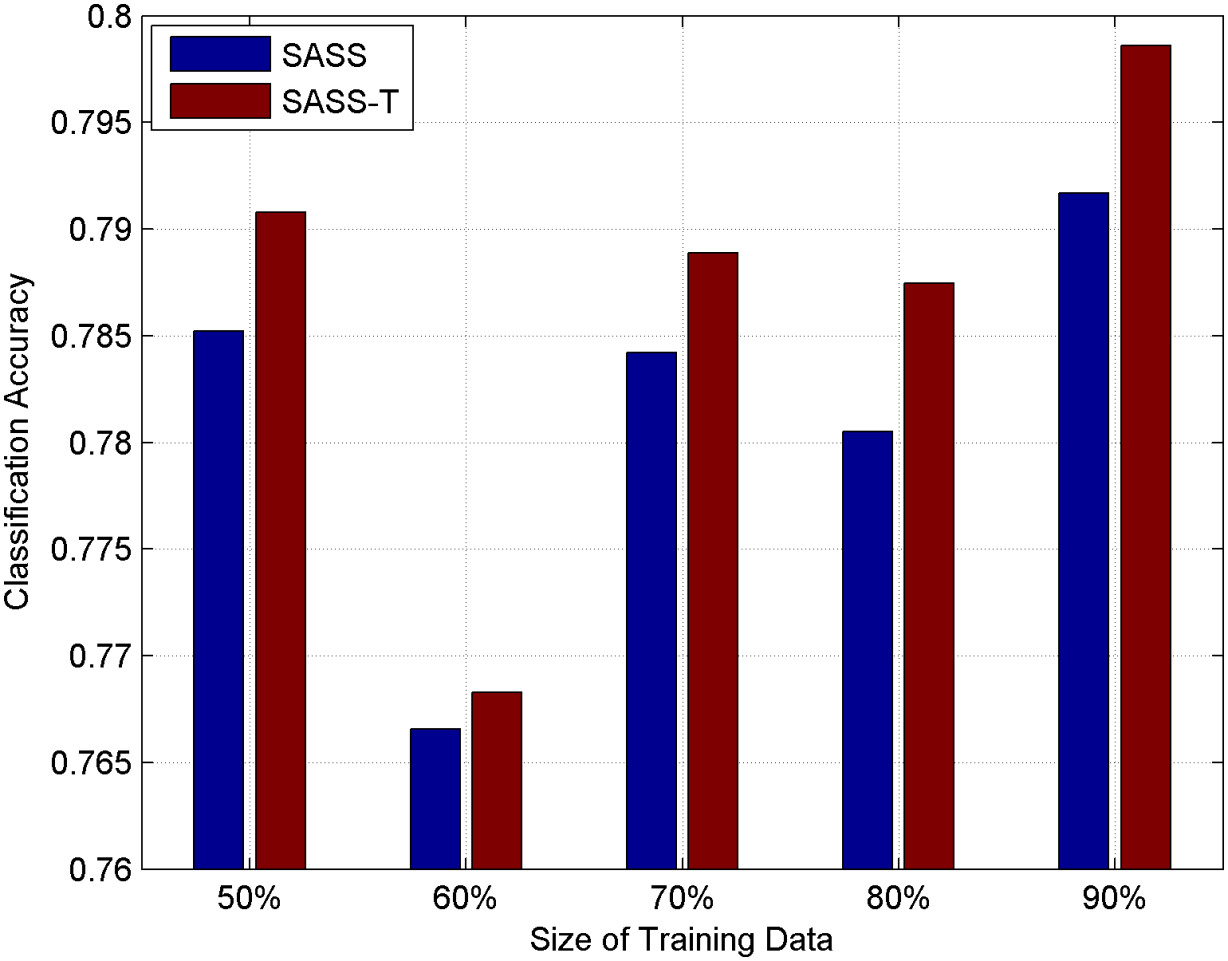
Classification accuracy on HCR with topic context.

**Fig 11:**
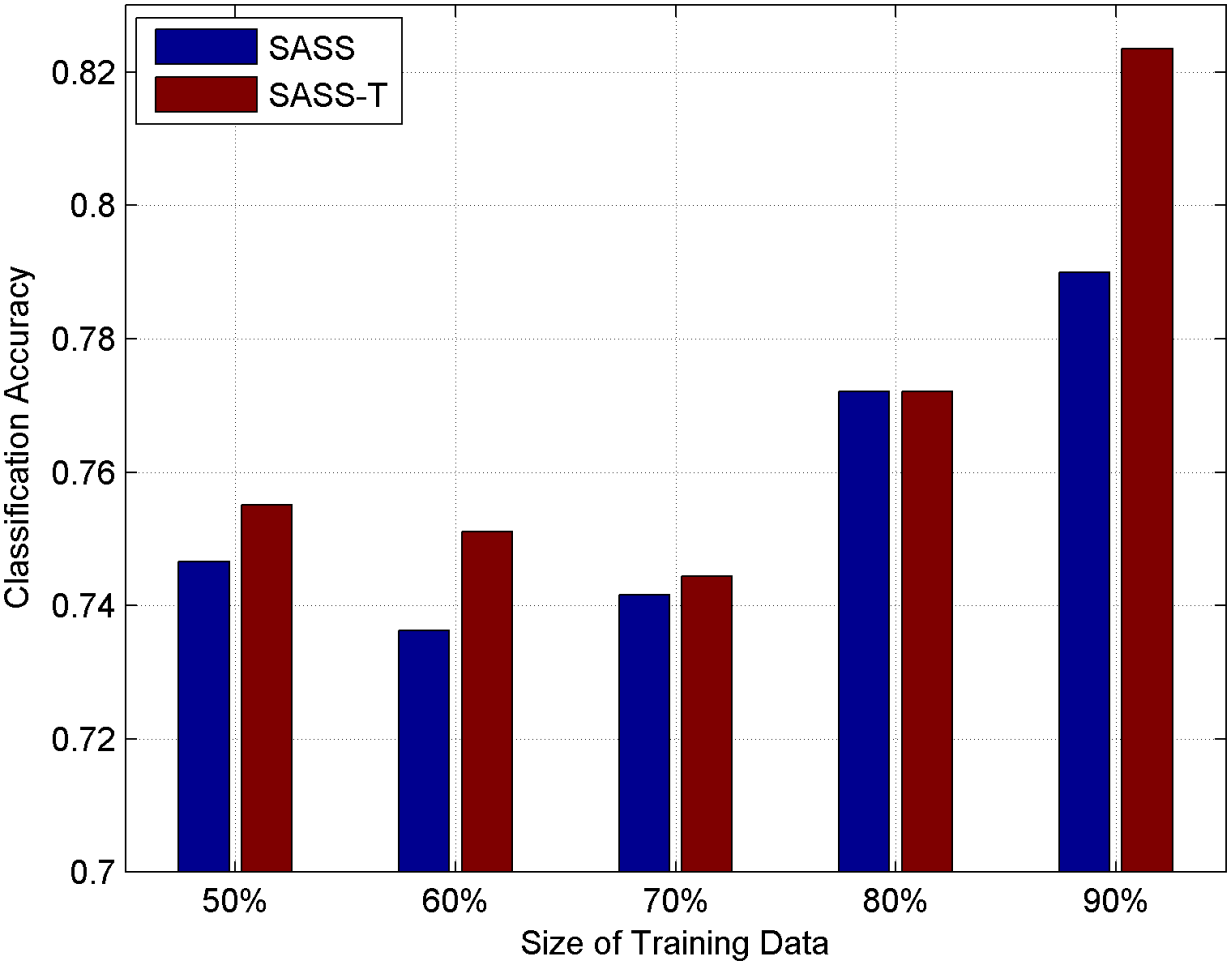
Classification accuracy on OMD with topic context.

In this subsection, we introduce topic context into our model and compare SASS with topic context (SASS-T) and SASS in different sizes of training data. Classification results are plotted in Figs 10 and 11 for HCR and OMD respectively. From the figures, we can see that adding topic context can improve the accuracy of microblog sentiment analysis to some extent. Compared with the traditional LS method, SASS-T can get the average percentage improvement by [22.4206% and 17.7539%] in HCR and OMD respectively, which is larger than SASS’s [21.61108% and 15.9152%]. T-tests are also applied in this subsection and there is also a significant improvement with the significance level 0.01 on both datasets. The results indicate the positive effect of using topic context to model the semantic relations between microblogs in microblog sentiment analysis. The reason why adding topics can improve the accuracy of sentiment analysis is that the opinions of a same person and similar users on the same topic usually consistent with each other.

### Parameter analysis

**Fig 12:**
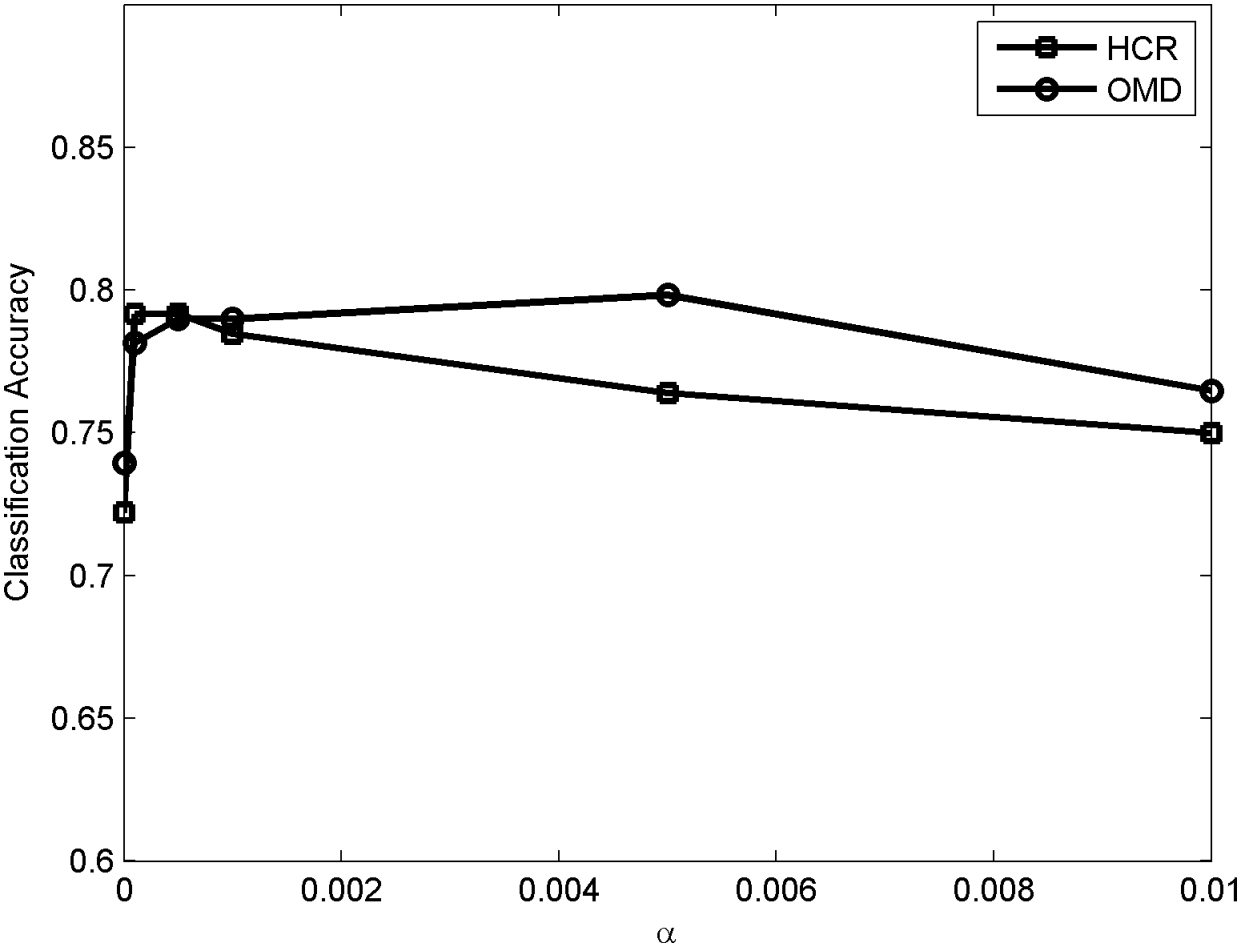
Sensitivity to hyper-parameter *α*.

**Fig 13:**
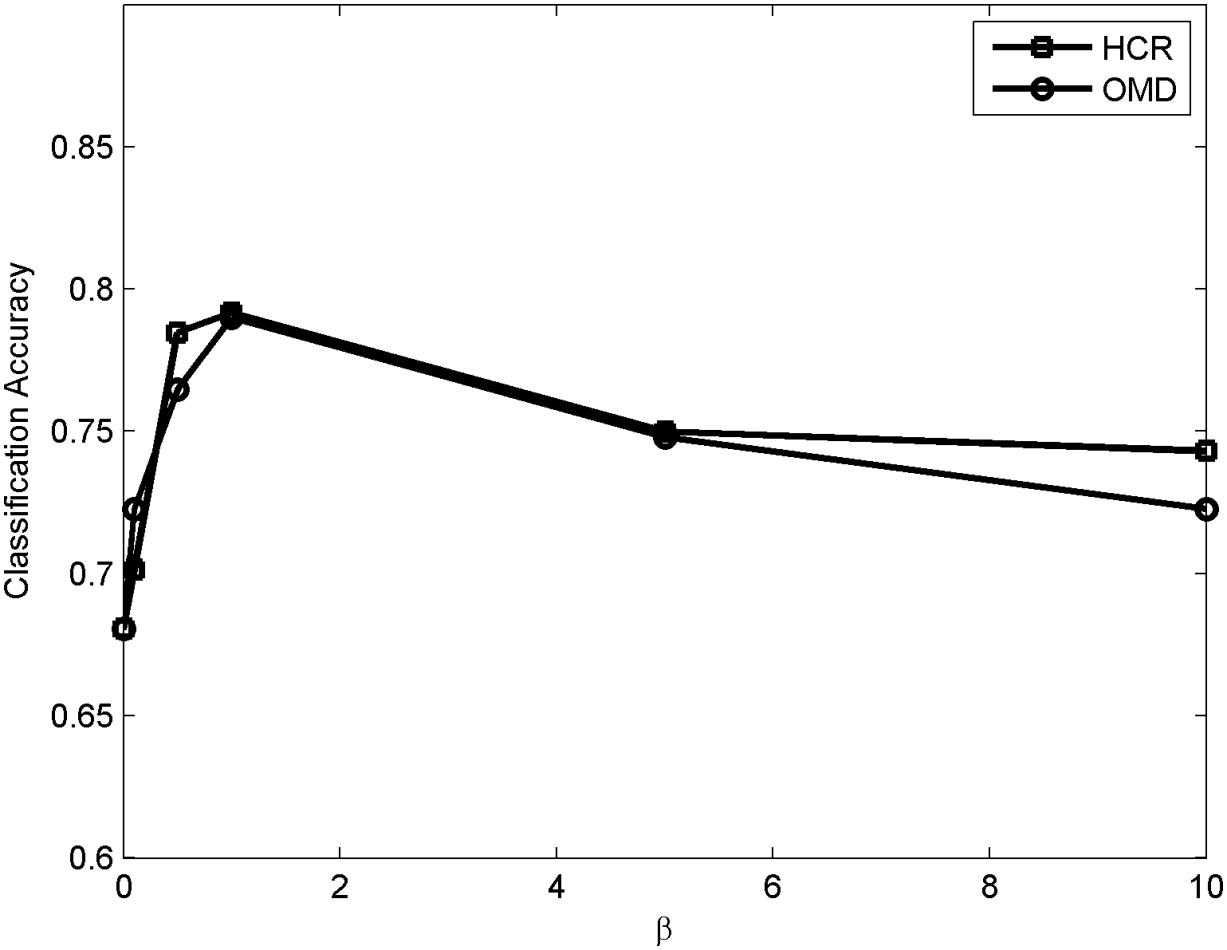
Sensitivity to hyper-parameter *β*.

In this subsection, we evaluate the effects of parameter selection of *α* and *β* on our method. We use 90% of data on both datasets which are randomly selected, and the left data are used for test. Fig 12 shows the effect of *α* in detail when *β* = 1. Obviously, the performance of SASS is not sensitive to the variation of *α*. When *α* is too small, social context is not fully used in the sentiment analysis. Thus, the performance increases as *α* increases from 0. However, when *α* is too large, the performance of the model mainly depends on social context so it becomes worse. Fig 13 shows the performance of SASS with the variation of *β* when *α* = 0.0005.

It is noted that when *β* is too large, the performance of our model goes down as it mainly relies on the sparse regularization and many features are filtered by the regularization. When *β* is too small, the sparse regularization is not fully used and many noises remain in the training set, so the accuracy of sentiment analysis also increases from 0. Besides, it is clear that the model is not very sensitive to the variation of *α* and *β* and it is an appealing property as it can save a lot of time to tune parameters.

## Conclusion and discussion

In this paper, we propose a new method which using social context to identify sentiment polarity. Inspired by sentimental consistency and emotional contagion, we take three kinds of context into account: user context, structure similarity context, and topic context. We introduce a measure to structure similarity, build structure similarity matrix. We also introduce topic context and build a topic context matrix. We add all these contexts into the model by using the Laplacian matrix of the graph constructed by the contexts. Experimental results show that structure similarity has a better performance than user direct relations. Besides, adding topic context is helpful for improving the accuracy of sentiment classification. Meanwhile, our method can be easily extended to other models such as semi-supervised classification model proposed by [50] and the structured model proposed by [51].

In this paper, we use Least Squares to model text information of microblogs. In future, we also want to extend Laplacian regularization to support vector machine (SVM) and maximum entropy model to see the differences between them. Deep learning methods have obtained a very good performance across many different NLP tasks recently, so we also want to study how to combine social context with deep learning models.
